# Survivorship care planning in gynecologic oncology—perspectives from patients, caregivers, and health care providers

**DOI:** 10.1007/s11764-018-0713-9

**Published:** 2018-09-12

**Authors:** Belle H. de Rooij, Teresa Hagan Thomas, Kathryn E. Post, Jane Flanagan, Nicole P. M. Ezendam, Jeffrey Peppercorn, Don S. Dizon

**Affiliations:** 1 Massachusetts General Hospital Cancer Center, Harvard Medical School, Boston, MA USA; 2 CoRPS - Center of Research on Psychology in Somatic diseases, Department of Medical and Clinical Psychology, Tilburg University, Tilburg, The Netherlands; 3 The Netherlands Comprehensive Cancer Organisation, Utrecht, The Netherlands; 4 University of Pittsburgh School of Nursing, Pittsburgh, PA USA; 5 Boston College William F. Connell School of Nursing, Boston, MA USA; 6 Lifespan Cancer Institute/Rhode Island Hospital, Alpert Medical School of Brown University, Providence, RI USA

**Keywords:** Cancer survivorship, Survivorship care, Survivorship care plan, Gynecologic cancer, Qualitative research

## Abstract

**Purpose:**

This qualitative study sought to describe the challenges following treatment and the preferences regarding survivorship care among patients treated for gynecological cancer, their caregivers, and health care providers.

**Methods:**

Between July and August 2017, in-depth semi-structured interviews regarding survivorship were conducted at a large academic hospital in the USA among patients who recently completed treatment (< 12 months) for a gynecological cancer (ovarian, endometrial, cervical, and vulvar) and their primary caregivers. A focus group was conducted among health care providers (oncologists, nurses, and fellows). Main themes were identified using descriptive content analysis.

**Results:**

A total of 30 individuals participated in this study (13 patients, 9 caregivers, 8 health care providers). Almost all participants reported a desire for more information on how to address survivorship needs, specifically as they related to side effects, follow-up schedule, and psychological assistance. Despite this uniformly identified need for more information, preferences for survivorship care planning differed across cancer types and individuals, with respect to content, timing, and mode of delivery. Health care providers expressed challenges in communicating with patients about survivorship, a desire to shift post-treatment conversations to the goal of improving quality of life as opposed to focusing on disease recurrence, and an unmet need for disease specific and individualized survivorship care planning.

**Conclusions:**

Patients, caregivers, and health care providers each expressed a need for gynecologic cancer-tailored survivorship care resources.

**Implications for Cancer Survivors:**

The variation of disease types and patient and caregiver needs may require multi-faceted, individualized survivorship care planning.

**Electronic supplementary material:**

The online version of this article (10.1007/s11764-018-0713-9) contains supplementary material, which is available to authorized users.

## Introduction

Each year, almost 106,000 women in the USA are diagnosed with a gynecological cancer [1]. Ovarian cancer remains the deadliest gynecological cancer, followed by vaginal, cervical, endometrial, and vulvar cancers. The estimated 5-year survival rates vary widely from 46% for women with ovarian cancer to over 80% for women with endometrial or vulvar malignancies [1]. Survival rates for gynecological cancers have slightly increased during the past decades, reflecting advances in treatment that ultimately help patients survive their disease [1].

Regardless of prognosis, a wide body of literature supports the notion that women treated for gynecological cancer experience a significant level of disease- and treatment-related symptoms that persist after the end of first-line treatment and greatly impact their long-term quality of life [2–7]. In addition, one of the most significant and overwhelming psychological concerns cancer survivors struggle to manage is the fear of cancer recurrence, which is associated with increased symptom burden, overwhelming anxiety, post-traumatic stress, and hopelessness [4, 6, 8–10].

The National Academy of Medicine (NAM) acknowledged that these factors are important in the ongoing care for cancer patients ending treatment, and in 2006 recommended that all cancer survivors receive a Survivorship Care Plan (SCP) [11]. SCPs typically contain written information on diagnosis, documentation of all treatments, short- and long term effects of the treatments, and recommendation for follow-up care [11]. To date, multiple randomized controlled trials evaluating the impact of SCPs among cancer patients [12–17], including gynecological cancer [13, 16, 18], have failed to demonstrate beneficial effects of SCPs on short- or long-term satisfaction with information provision and care, quality of life, or distress. These outcomes suggest that patients with gynecological cancer may not receive the intended benefits of an SCP as proposed by the NAM without further refinement and evaluation of these interventions [19].

Part of the disconnect between the prominent calls for SCPs as a self-evident beneficial intervention and the lack on strong evidence supporting SCPs in their current format may stem from a failure to adequately tailor these interventions to the needs of specific cancer patient populations. Additionally, there may be a need to further adapt the intervention to the needs and preferences of the individual patient. Given the lack of evidence to support existing SCP models in the setting of gynecologic oncology, we sought to describe the perspectives of patients with gynecological cancer, as well as their caregivers and health care providers (HCPs). This may provide insights into the unmet needs of patients and their caregivers as well as challenges to the health care team and identify opportunities for effective intervention through SCPs or other aspects of survivorship care.

The aim of the current study is to describe the (1) challenges following treatment and (2) the preferences regarding survivorship care among patients treated for gynecological cancer, their caregivers, and HCPs.

## Methods

### Design

This study employed an open-ended qualitative descriptive design including in-depth semi-structured interviews among three stakeholder groups: patients with a gynecological cancer, their caregivers, and gynecologic oncology HCPs with whom we conducted a focus group. The study protocol was approved by the Dana-Farber/Harvard Cancer Center Institutional Review Board.

### Participants and recruitment

Patients older than 18, able to read and respond in English, that completed treatment for any type of gynecological cancer within the past 12 months, were eligible to participate in the study. The study team reviewed upcoming clinic schedules for eligible patients and invited those patients to participate during a scheduled outpatient clinic visit at the Cancer Center, or were invited by phone. Patients were asked to identify their primary caregiver defined as a spouse, family member, or friend who provides care and support to the patient. Caregivers were introduced to the study at the Cancer Center, or by phone when not present at the Cancer Center. After informed consent, telephone or in-person interviews with patients and caregivers were scheduled at a time convenient for them. HCPs that primarily provide care for patients with gynecologic cancer (gynecologic oncolgists, medical oncologists, gynecologic oncology fellows, and nurse practitioners) were invited to participate in a focus group interview during a regular gynecologic oncology staff meeting. Informed consent was obtained at the beginning of the meeting.

### Data collection

Telephone or in-person interviews with patients and caregivers and the HCP focus group were digitally recorded. Audio-recordings were transcribed verbatim to text using TranscribeMe.com. In addition, demographic questionnaires were completed by patients and caregivers. Measures included age, sex, ethnicity, education, and employment. Clinical patient data was extracted from each patient’s electronic medical record.

### Interview guide

Semi-structured qualitative interview guides were developed by members of the study team. We purposefully included open-ended questions to determine patient and caregiver needs a priori with minimal predetermined categories of survivorship care planning topics. While the patient and caregiver questionnaire guides included similar items tailored for each group, separate questionnaire guides were developed for HCPs. The interview guides were discussed and refined by study team members resulting in a list of questions and follow-up probes for each group.

### Data analysis

Data transcripts were coded in NVivo 11 (QSR International) using descriptive content analysis techniques. The initial coding scheme for patient and caregiver interviews was based on the first three patient interviews and iteratively refined and expanded while reviewing additional interviews. Two study researchers (THT and BHR) generated the categories independently through a close reading of the transcript texts, jointly comparing their categories, reviewing any discrepancies and disagreements, and resolving discrepancies through consensus. We continued reviewing coding until saturation was achieved and no new category themes emerged. After developing a comprehensive list of categories, we then summarized and classified the categories into higher-order themes. To ensure consistency between themes, categories, and the raw data, we selected representative quotations of each theme to illustrate its meaning and assist with data interpretation. Codebooks were developed for patient interviews first and applied to the caregivers’ interviews after determining similar content between these interviews. The HCP focus group was coded separately due to their distinct perspective and ideas discussed. We calculated the frequency of specific categories and compare these to patients’ and caregivers’ responses. Based on emerging categories indicating differences in type of gynecological cancer, we also decided to compare the perspectives of patients and caregivers with ovarian cancer versus other gynecological cancer types. As a qualitative study, we focused our comparisons on basic descriptive statistics and did not use our quantified data to statistically test group differences to avoid over-simplifying our qualitative exploratory data.

## Results

In total, we had 30 participants included in this study (13 patients, 9 caregivers, and 8 HCPs). Five patients that were approached did not want to participate (no time/busy or did not want to be reminded of their cancer) and one patient was lost to follow-up. Four caregivers identified by patients chose not to participate in the study. Only one patient had an in-person interview, and all other patients and caregivers preferred telephone interviews. Interviews for patient and caregiver participants lasted 30–40 min. The focus group interview of the providers was 35 min.

### Participant characteristics

Table 1 describes patient and caregiver characteristics. Patients (*n* = 13) represented various gynecological cancer types, including ovarian (*n* = 5), endometrial (*n* = 4), cervical (*n* = 2), fallopian tube (*n* = 1), and vulvar (*n* = 1), had an average age of 63, were predominantly white (92%), unemployed at the time of the interview (62%), and completed treatment 6 months before the interview. Caregivers of patients (*n* = 9) were mostly the patient’s spouse (*n* = 6), had an average age of 59, were predominantly male (78%), white (100%), and employed at the time of the interview (56%). The HCP focus group (*n* = 8) included gynecologic oncolgists (*n* = 2), gynecologic oncology fellows (*n* = 3), a medical oncologist (*n* = 1), a radiation oncologist (*n* = 1), and a nurse practitioner (*n* = 1), and were predominantly female (*n* = 5, 63%).

**Table 1:** Patient and caregiver characteristics

	Patients			Caregivers		
	Total (*N* = 13)	Ovarian cancer (*n* = 6)	Non-ovarian cancer (*n* = 7)	Total (*N* = 9)	Ovarian cancer (*n* = 5)	Non-ovarian cancer (*n* = 4)
Age, *M* (min-max)	63.1 (48–71)	63.0 (48–71)	61.7 (51–75)	58.7 (34–73)	60.4 (34–73)	56.5 (37–79)
Sex						
Male	0 (0)	0 (0)	0 (0)	7 (78)	4 (80)	3 (75)
Female	100 (100)	100 (100)	100 (100)	2 (22)	1 (20)	1 (25)
Ethnicity						
White	12 (92)	6 (100)	6 (86)	100 (100)	100 (100)	100 (100)
Asian	1 (8)	0 (0)	1 (14)	0 (0)	0 (0)	0 (0)
Educational level^a^						
High school diploma or 2-year/associate’s degree	3 (23)	2 (33)	1 (14)	1 (11)	1 (20)	0 (0)
4-year/ bachelor’s degree	3 (23)	1 (17)	2 (28)	3 (33)	1 (20)	1 (25)
Graduate/professional degree	4 (31)	3 (50)	1 (14)	3 (33)	3 (60)	1 (25)
Unknown	3 (23)	0 (0)	3 (43)	2 (22)	0 (0)	2 (50)
Currently employed						
Yes	3 (23)	1 (17)	2 (28)	5 (56)	2 (40)	1 (25)
No	8 (62)	5 (83)	3 (43)	2 (22)	3 (60)	1 (25)
Unknown	2 (15)	0 (0)	2 (28)	2 (22)	0 (0)	2 (50)
Patient clinical characteristics						
Cancer type, *N* (%)						
Ovarian	6 (46)	6 (100)	N/A	7 (55)	5 (100)	N/A
Endometrial	4 (31)	N/A	4 (57)	1 (11)	N/A	1 (25)
Cervical	2 (15)	N/A	2 (29)	2 (22)	N/A	2 (50)
Vulvar	1 (8)	N/A	1 (14)	1 (11)	N/A	1 (25)
Cancer stage, *N* (%)						
I	3 (23)	0 (0)	3 (23)	0 (0)	0 (0)	0 (0)
II	4 (31)	2 (33)	2 (28)	4 (44)	2 (40)	2 (50)
III	2 (15)	3 (50)	1 (14)	1 (11)	2 (60)	0 (0)
IV	3 (23)	1 (17)	2 (28)	3 (33)	1 (20)	1 (25)
Unstaged	1 (8)	0 (0)	1 (14)	1 (11)	0 (0)	1 (25)
Treatment type, *N* (%)						
Surgery only	2 (15)	0 (0)	2 (28)	0 (0)	0 (0)	0 (0)
Chemotherapy	6 (46)	1 (83)	1 (17)	5 (55)	4 (80)	1 (25)
Chemo + radiotherapy	5 (38)	1 (17)	4 (57)	4 (44)	1 (20)	3 (75)
Months since end of treatment, *M* (min-max)	6.4 (1–11)	5.2 (1–10)	7.4 (2–11)	5.9 (1–11)	8.0 (2–9)	4.2 (1–10)

*N/A* not available

### Perspectives of patients and caregivers

The major categories found in patient and caregiver interviews were (1) symptoms and concerns, (2) fear of recurrence, (3) information, (4) needs, (5) satisfaction with care, (6) self-management, and coping (7) preferences for survivorship care planning. Illustrative quotations are presented below and additional quotations are stated in Table 2.

**Table 2:** Themes identified and exemplary quotes of patients and caregivers

Theme	Description	Patients’ quotes	Caregivers’ quotes
Symptoms and concerns	Distressing symptoms (chemo-brain, constipation and diarrhea, hair loss, neuropathy, nausea, pain), mood (anxiety, depression), recovering from the cancer and treatment	“A roller coaster, you’re like, some days you’re fine. And then other days, I get myself getting a little depressed, and anxious, and overwhelmed with the thought that I had cancer. So I don’t know. Maybe they could be some sort of help in that way.” (Patient 3, fallopian tube cancer, stage IIB) “In my mind, I was thinking that when chemo was done, I was going to be fine. I never experienced the side effects during my chemo but the people do. All the side effects happened at the end so-- which was bit alarming to me because I thought, well, maybe I had escaped all the side effects. But I hadn’t.” (Patient 7, ovarian cancer, stage IIIB)	“So the biggest concern is when is this all going to go away? When is it all going to be over and she’ll just be back to normal, I guess, is the biggest concern.” (Caregiver 14, cervical cancer, stage IIB) “It’s very reassuring to hear them tell you what to expect and that the symptoms that she’s going through are just normal and they’re going to get better and it’s-- that’s very reassuring to hear it from somebody live than to read about it and just to get notes on it, so.” (Caregiver 14, cervical cancer, stage IIB)
Fear of recurrence		“…I’d rather not dwell on something else. I don’t think that’s a productive use of time.” (Patient 6, endometrial cancer, stage IA)	“But I suppose it’s always going to be something in the back of my mind, I’m just going to think about probably more than 50% of cancer patients get it back at some point in their life. So yeah, it’s, again, probably somewhat concerned. Does it affect my day-to-day life or anything like that? No.” (Caregiver 4, endometrial cancer, stage IIIA; and ovarian cancer stage unknown)) “I know it is going to come back…. It affects my attitude toward my future.” (Caregiver 7, ovarian cancer, stage IIIB)
Information	Wanting information on things to look out for, not wanting too much information, not wanting to be scared		“If there’s anything I can do to detect the symptoms so early on, regardless of how minute or simple it may be, and how silly it might prompt a doctor’s appointment, I want to know about it.” (Caregiver 1, ovarian cancer, stage I), stage IIIB)
Needs	Need for information (complementary treatments, contact information/referral, follow-up plan, lifestyle, recurrence and prevention, side effects and treating symptoms, support groups and peer support)	“Nobody’s told me, so I’m assuming that everything’s taken care of. And she’s [oncology provider] telling me that … it can come back. Well, where is it going to come back? What do I look for? I mean, I’m going down there every six months to be checked, what is she checking? I don’t know.” (Patient 2, endometrial cancer, stage IA) “I would like to stay with [cancer hospital] as part of my survivorship care. I do not choose to transition over to my primary care…. And I’m hoping that [cancer hospital] will let me do that. And my primary care, I like, but I don’t have a very strong allegiance to the hospital community that they represent or that they are affiliated with. I just don’t care for their quality of care.” (Patient 7, ovarian cancer, stage IIIB) “I’ve got side effects that I’m trying to get help with, and I’ve got three different doctors, and I’m talking to each of the doctors, and it seems to be nobody’s responsibility to assist me with these issues. I don’t know the primary person that I should call. There’s a definite disconnect between the doctors.” (Patient 13, cervical cancer, stage IIA)	“What can we do? I know there’s tons of stuff we can do to help prevent cancer. Just by eating a cleaner diet, less processed food. So I want to know about those things. Nutritional issues, supplements to help keep it from coming back, lifestyle changes. What are the signs and symptoms to look for if you’re not feeling well? Is it a sign or a symptom that is going to signal something is going on again? Those things. I mean, aside from the general if you feel pain, if you have discharge, if you’re bleeding. Is there something else? Maybe something more subtle?” (Caregiver 1, ovarian cancer, stage IV) “A lot of those symptoms can be symptoms of something else as well. So if you’ve got a backache or you’ve got nausea, you’re tired, or whatever, it might be from cancer and it might not. So I don’t know if-- probably no way to really help differentiate between what’s bad and what’s attributable to something else.” (Caregiver 10, endometrial cancer, stage IV) “I can’t really say that the team didn’t say this could happen because I’m not sure that-- I don’t know anything that could really prepare you for what happened and I think that what happened is-- my impression is that it just-- what happens and it can be really different all the time.” (Caregiver 11, vulvar cancer, stage unknown)
Satisfaction with care	Satisfaction with follow-up care, information and resources of oncology team, trust in oncology health care providers	“The other part of our conversation was about if the cancer comes back and … he assured me that, just because I am not being seen every three months, doesn’t mean that they’re not paying attention to my needs during this time.” (Patient 8, ovarian cancer, stage IIIC) “As the treatments ended, there was a lack of understanding of what happens next. There wasn’t enough information given to me. At the beginning of the treatment, there’s a lot of information. This is how we all proceed. At the end of the treatment, there was no-- I was never told how often my visits would be, what I needed to look out for. It was just like last visit, and that was it. And walked away, and I was more confused at the end than I was at the beginning.” (Patient 13, cervical cancer, stage IIA)	“I’m assuming that the medical profession has worked out these numbers for the first year, you do a certain thing, for the next years, you do other things, and I’m comfortable with that.” (Caregiver 12, ovarian cancer, stage IIB) “I assume they go for a one-hour appointment, and they spend ten minutes. Fine if that works. But [patient] has a lot of questions. And I can always sense that the provider, very politely, is watching their watch and trying to get out of the room. Fine. This is treatment. Let’s get it done. But when you get the survivorship, I would think that it would be a little better to just let the person have their half hour or hour, whatever it takes, that they’re comfortable.” (Caregiver 7, ovarian cancer, stage IIIB) “It’s just a really poor lack of continuity in care and results. Dr. [name] didn’t want to give her pain medication because he couldn’t understand why she was having pain, and then she came back and then she was at [hospital] trying to get pain management. They wouldn’t give her any pills, because they never saw the scans. So she kind of just ping-ponged back and forth between these two doctors, and all she wanted was pain management.” (Caregiver 13, cervical cancer, stage IIA)
Self-management and coping	Searching for health information online, support from others, spiritual coping, being optimistic, doing everything you can	“I would say that I’m probably more hyper-vigilant around if something doesn’t feel right that I investigate.” (Patient 11, vulvar cancer, stage unknown) “I just have to keep praying and be hopeful that the kind of treatment I had, (…) has worked and will give me five years cancer free. That’s what I’m hoping for.” (Patient 7, ovarian cancer, stage IIIB) “So I asked a lot of questions. I talked to a lot of people. I called friends that were nurses, nurse practitioners, people that have gone through it. I asked them questions. I told my family, and they contacted people.” (Patient 4, endometrial cancer, stage IIIA; and ovarian cancer stage unknown)	“I want to make sure that I’m not missing anything. I know they do studies. She’s already been told she can participate in studies. I want to have keen and constant awareness of what’s going on in that area.” (Caregiver 7, ovarian cancer, stage IIIB) “Knowing other people were going through it was very empowering, that you weren’t the only one. That a lot of the things that you were feeling, they were feeling, and that there were a bunch of people trying to get through this together, as opposed to an individual.” (Caregiver 3, fallopian tube, IIB) “So we used that as well as just looking stuff up on the internet. But the problem with the internet it’s like anything you look up, you could look up ten sites and have ten different opinions.” (Caregiver 4, endometrial cancer, stage IIIA; and ovarian cancer stage unknown)) “We’d remained pretty optimistic, given her performance and sort of focusing on the idea that the-- I think somebody has to be in the 10% that survive. And we were focusing on that. When the blood work came back that sort of blew that out of the water. So I’m sort of reassessing at this point.” (Caregiver 10, endometrial cancer, stage IV)
Survivorship care planning	Preferences for survivorship care planning regarding content, mode of delivery, timing and people involved, personalized survivorship care planning	“I think it’s just like if something’s abnormal. I need to say something. I think it starts with me. No one’s going to know unless I say something, and the doctors, I think they’re pretty much on the ball, at least the ones I’ve worked with.” (Patient 6, endometrial cancer, stage IA) “I’d like it as soon as possible, within the next six months, three months. The document would certainly be something that would be created by hopefully myself, my oncologist, and my husband. And I wouldn’t mind having an infusion nurse there with us. Because the nursing staff has different perspective on things than oncologists themselves.” (Patient 7, ovarian cancer, stage IIIB) “Sometimes when you’re sitting in the doctor’s office, which is why my husband always comes with me, I hear things differently than he might hear something or I might not hear something at all or just think it wasn’t important and he will say, “Well, it was kind of important,” or, “No, that wasn’t really what I think the doctor meant.” (Patient 14, cervical cancer, stage IIB) “Any updates in the therapies or studies that would give hope to an ovarian cancer survivor, if they could be updated on the internet and an email sent to the patient stating, ‘This is what we’ve found, this is our most recent findings, and this is how it would impact you.’” (Patient 8, ovarian cancer, stage IIIC)	“I think that at first post-treatment appointment, [survivorship care planning] should be a conversation. And it should be on paper and then follow up with maybe an electronic version of any update, what have you.” (Caregiver 1, ovarian cancer, stage IV) “I think, obviously, the patient, and hopefully, the caregiver could be there [for survivorship care planning]. And that’s who should-- certainly, the patient needs to receive it, and it would be nice if the caregiver got the same thing. But I still think it would be useful to be in a group setting.” (Caregiver 3, fallopian tube, stage IIB) “The things that I’d like to know about are the things that are the most important things to look for. But … I’m not sure that I would even be qualified or [patient] would be qualified to evaluate certain symptoms, right? Without real medical training.” (Caregiver 11, vulvar cancer, stage unknown) “I think it should be throughout the course of treatment. Like I said, it will always probably be a snapshot of where you are in treatment and what is going on. As the treatment progresses, I imagine that some of this information will change or emphasis will be shifted.” (Caregiver 12, ovarian cancer, stage IIB) “I think personalized to a certain degree. I wouldn’t expect someone to go and create the [caregiver] and [patient] website. I wouldn’t expect there to be someone who would go through, but I would expect there to be some kind of level of, ‘Okay. Here are the resources that are pertinent.’” (Caregiver 11, vulvar cancer, stage unknown) “I would certainly want it updated. I would like to receive an initial with all updated information at the time of the appointment. If It’s anything new developed in the meantime, any new significant treatments, anything based on study, yeah, that should be sent to people via mail, email, or both.” (Caregiver 1, ovarian cancer, stage IV)

#### Symptoms and concerns

Patient symptoms causing distress were described by the majority of both patients and caregivers (10/13 patients; 9/9 caregivers). Pain (4/13 patients; 2/9caregivers), neuropathy (3/13 patients; 2/9 caregivers), fatigue (3/13 patients, 1/9 caregivers) and anxiety/depression (2/13 patients; 4/9 caregivers) were the most commonly discussed distressing symptoms. One patient noted that managing her symptoms after treatment ended was particularly challenging:

Post-treatment… that was the hardest time during the whole process because there were a multitude of side-effects that I was dealing with that … I didn’t have enough information about …I just wasn’t reassured enough that it was going to get better. Or maybe I was unable to absorb that it was going to get better. (Patient 11, vulvar cancer, stage unknown).

Almost half of patients (6/13) expressed that they had limited or no post-treatment symptoms, indicating that whatever symptoms they did experience were not distressing.

While some caregivers reported a similar desire for reassurance that physical symptoms would subside post-treatment, they reported many more concerns about dealing with the emotional adjustment. For example, one caregiver described his lack of training in medical symptoms and concern about his ability to emotionally support his wife:

I’m not too concerned with the physical stuff. I can deal with that. Her feet don’t work. Her hands don’t work. And she’s had a profound hearing loss. I have no training background or anything in how to assist with that. But she’ll say, ‘Can you open this for me?’ … those easy things…. I’m more concerned with the emotional support and maybe being sensitive, those types of things. (Caregiver 7, Fallopian tube cancer, stage IIB).

#### Fear of recurrence

Fear of recurrence was common among both patients and caregivers (9/13 patients; 8/9 caregivers). Some patients reported overwhelming preoccupation with the chance that their cancer could return:

I’ve had a lot of anxiety over it. Like if I get a pain, right away, my head goes to the worst-case scenario. So the fact that I had the cancer, it makes me more anxious about thinking that I could get it somewhere else. (Patient 3, fallopian tube cancer, stage IIB).

Of interest, despite the majority confirming they experienced fear of recurrence, most also noted they were not preoccupied with this fear (10/13 patients; 2/9 caregivers).

#### Informational needs

Informational needs were reported by both caregivers and patients and included possible signs or symptoms of recurrence (11/13 patients; 8/9 caregivers), management of side effects (7/13 patients; 6/9 caregivers), contact information for care providers or sources of specialized services (6/13 patients; 2/9 caregivers), symptom management (4/13 patients, 4/9 caregivers), and methods to reduce risk of recurrence or new cancers (no patients; 5/9 caregivers).

#### Self-management and coping

Patients and caregivers wanted to know what the range of expected ongoing issues might be so that they could make informed decisions about when to contact their oncology HCP. Patients saw this as a way to self-manage and control their health:

I am the best steward for my body. I’m the one that looks at it and feels it every day….I need to have as much education as I can have so that I can take care of my body (Patient 8, ovarian cancer, stage IIIC).

Caregivers felt similar desires:

That would be my job to decide or not. But no, I don’t want the medical providers deciding that. I want to know everything (Caregiver 7, ovarian cancer, stage IIIB).

Some patients did not want to be scared by the post-treatment side effects:

I don’t like to get more information than what I really need to know. I don’t want to scare myself…. I was going through this with just being calm and see what happens (Patient 2, endometrial cancer, IA).

#### Satisfaction with care

Although the majority reported satisfaction with the current informational resources they received from their oncology HCP (11/13 patients; 9/9 caregivers), almost all (12/13 patients; 9/9 caregivers) expressed a need for supplemental information to address their remaining issues and ongoing concerns. Most patients and caregivers reported feeling like they could contact their oncology HCP whenever they needed help:

I’m not that concerned because I know that if something comes up and I’m unsure, I can call them and see them, or I can call them and ask them (Patient 4, endometrial cancer, stage IIIA; and ovarian cancer, stage unknown).

#### Survivorship care planning

Patients and caregivers mainly prefered to receive an SCP in written form (8/13 patients; 5/9 caregivers) though the majority noted that both written and online were acceptable (7/13 patients; 6/9 caregivers). Most wanted the SCP to be updated overtime (9/13 patients; 6/9 caregivers), and many wanted to receive the SCP at first follow-up visit (5/13 patients; 5/9 caregivers). Some did not think that a SCP would be applicable to them (3/13 patients; 1/9% caregivers) because they received minimal treatment:

…They were very thorough with telling me everything that happened. Maybe it might have been different if I was getting further treatment like the chemo or radiation. I think you would want to know more information about that and how this is going to work or, I don’t really know. (Patient 2, endometrial cancer, stage IA).

While patients and caregivers varied in their preferences for the ideal content and timing of SCPs, most described their choices as based on their evolving state of health. Therefore, single review of treatment and care plan at the completion of initial therapy as a one time SCP to address survivorship concerns does not appear to be sufficient. Patients and caregivers wanted information when it would be immediately relevant to their health and well-being at multiple points across the disease trajectory:

It all depends on my state of health. If I am very sick, I don’t think I even need the information, but if I’m starting with symptoms, as soon as possible. So we, myself and my care team, will have that plan in motion for treatment. (Patient 1, ovarian cancer, stage IV).

### Ovarian versus non-ovarian cancer

Compared to patients with non-ovarian cancer types (*n* = 7), patients with ovarian cancer (*n* = 6) more often reported mood problems such as anxiety and depression (2/6 ovarian; 0/7 non-ovarian) and chemo-brain (2/6 ovarian; 0/7 non-ovarian), while non-ovarian cancer patients more often reported having no or limited symptoms (2/6 ovarian; 4/7 non-ovarian). Coping strategies of ovarian cancer patients were more often spiritual (4/6 ovarian; 1/7 non-ovarian) and trying to be optimistic (4/6 ovarian; 1/7 non-ovarian):

I don’t look back. Right now, I don’t have cancer and I choose not to think that it’s coming back. I’m very positive. I live for today and that’s how I manage. I don’t know about anybody else but that’s my attitude. (Patient 1, ovarian cancer, stage IV).

With regard to survivorship care planning, both ovarian cancer patients and caregivers preferred to receive written information (6/6 ovarian patients; 2/7non-ovarian patients; 4/5 ovarian caregivers; 1/4 non-ovarian caregivers). Many of the patients with other types of gynecological cancers—but none of the patients with ovarian cancer—reported that they were not interested in a SCP because it was not relevant to their situation (0/6 ovarian; 3/7 non-ovarian).

I think this question [about SCPs] is more for people that have been through a lot more than what I have been through. (Patient 2, endometrial cancer, Stage IA).

### Perspectives of health care providers

The HCP focus group included a detailed discussion on the challenges they encounter while communicating about survivorship. Illustrative quotations are presented below and additional quotes are stated in Table 3. A major barrier to communication was feeling an underlying tension between being direct about the likelihood of a recurrence without stripping away the patient’s ability to enjoy life. They reported a reluctance to “scare” patients with information about recurrence and ongoing health issues as a way to help patients focus on enhancing their quality of life:

There’s always this really inherent tension in that visit, between stating that [the cancer is incurable] again, and taking away the reprieve that they’re about to have.... The tension between being honest and being cruel, or being misleading. And it’s very complex, and the language is very complex.... So it’s a tight dance (Provider 4, gynecologic oncologist).

Another challenge to communication was prognosis. For patients who were likely to experience a recurrence (e.g., patients with ovarian cancer), providers desired to reinforce that patients should live life in spite of fear and uncertainty:

It’s a question of how do we convey to patients that the time that they have in remission is precious and important? And they shouldn’t delay life events thinking that they’re going to have a really long time to sort of get to that later (Provider 1, gynecologic oncologist).

Despite the information available, HCPs felt they continued to struggle to find necessary resources for patients. They expressed the need for survivorship-care resources to facilitate and support conversations about what to expect after treatment including a follow-up plan. The examples described by one provider describe the extent and details of the resources providers wanted to provide their patients:

I think it would be nice just to have resources about how to get back to your normal life. So what to do if you’re depressed or anxious, or how to get sexual function back, or interest, or exercise. So, things not just about the cancer, but how can we get back to your life and living with the cancer (Provider 6, gynecologic oncology fellow).

Providers also expressed that they want tailored and disease-specific SCPs to assist with difficult conversations, particularly referring to ovarian cancer as being different from other gynecological cancers:

Because you want to celebrate the win and not tell them that we’re going to run out of runway (Provider 1, gynecologic oncologist).

However, the main barrier to providing a SCP to patients identified by providers was lack of time. In addition to concerns over time to develop and present a SCP, some worried that providing a SCP might identify needs or open up conversations that providers could not address during the visit. Gynecologic oncologists preferred to have a medical doctor or other member of the gynecological cancer team provide an SCP, but some felt that this could be provided by a dedicated survivorship specialist as opposed to no one providing SCPs.

**Table 3:** Themes identified and exemplary quotes of health care providers

Theme	Description	Health care providers’ quotes
Challenges in post-treatment care	Challenges in communicating about survivorship, struggling to find necessary resources for patients, uncertainty about recurrence, not wanting to scare patients/improve quality of life	“I think I use it as a sort of metric about my degree of burnout. If I’m looking at the end-game for them, and they’re depressed about the potentially bad outcome, I feel like I’m a bit more burned-out. Whereas, if I’m celebrating with them now, I’m-- sort of feel like I understand the big picture, but where are they at now.” (Provider 2, medical oncologist) “Helping people be able to use that good time that they have because I know that ovarian cancer patients actually spend a lot of that time just worrying and freaking out. And if care plan can help with that, but I’d be hopeful it might. Whereas for early stage in endometrial cancer patients, who we tell, ‘You’re probably cured,’ I would actually want the care plan to be a little bit different and I actually want more there to be, like ‘If you have X kind of symptoms, or bleeding, or whatever, please give us a call.’ So I think, to me, I’d want them to be really shaped to what the general trajectory is that diseases tend to be.” (Provider 7, gynecologic oncology fellow)
Need for survivorship care plan and resources	Need for SCP (most common issues/percentages, reassurance, sexual health, support groups, diet, exercise, attitude, how to get back to normal, what to expect, follow-up plan), written information as supplement to conversation, referencing what to look out for and when to come back, disease-specific SCPs	“I don’t think I call it the survivorship plan. I think we just come up with a strategy for how they’re going to move forward with or without their cancer. And we talk about what’s sort of important.” (Provider 1, gynecologic oncologist) “[Patients] seem to think that they’re the only ones going through this process and they feel alone. And I never knew any of the resources to hook them up with. Like are there support groups out there? What are the resources they have so they don’t feel so alone and can go forward in the survivorship period of their lives.” (Provider 5, gynecologic oncology fellow) “if we had more of a standardized thing that we knew, oh, 80% of people have this, da, da, da, da, and you could kind of run through that check off and then have the immediate thing that they needed to get plugged into.” (Provider 1, gynecologic oncologist)
Barriers to providing survivorship care plans	Barrier of time, not wanting to open up difficult needs, who should provide SCPs (oncologist, nurse, anybody), logistics of providing SCPs, standardized list of prompts/screening tools	“What if you ask somebody and they go to pieces in front of you, and then you have like a whole new thing and you don’t have the ability to unpack it for an hour and a half. It’s really hard. So how do you do that? And how do you make them feel like you’ve heard them?” (Provider 1, gynecologic oncologist)

## Discussion

This study reports participants’ self-identified concerns and preferences for survivorship care. Findings indicate that patients with a gynecological cancer and their caregivers have needs and ongoing issues after treatment, such as side effects and psychological distress, and that they desire information on how to better address these needs. Preferences for survivorship care largely differ across cancer types and individuals, with respect to content, timing, and mode of delivery and reflect the need for disease-specific, tailored SCPs and follow-up care to support care to the diverse group of gynecological cancer survivors. Our results contribute to the ongoing discussion about effective and efficient means to support survivorship care planning in gynecologic oncology, further highlighting the fact that “one size fits all” approaches are unlikely to be successful, and individualized assessment and care planning is needed.

Issues, concerns, and symptoms most often discussed in our study are similar to previous work and include pain, neuropathy, fatigue, and mood problems such as anxiety, depression, and fear of recurrence [2–7]. As reported in previous literature, ovarian cancer patients more often described mood problems and fear of recurrence or progression compared to non-ovarian cancer patients [20]. As a result, compared to non-ovarian cancer patients, ovarian cancer patients more often expressed a need for contact information or referral for someone to help with these concerns.

Caregivers in our study reported similar perspectives as patients, but with several notable exceptions including more frequent endorsement of being afraid of a cancer recurrence or disease progression and wanting to learn health promotion strategies. These results complement growing literature describing the changing and often increasing needs of caregivers of individuals with gynecological cancer [21, 22]. For example, Stafford and Judd found that caregivers’ unmet needs were a key predictor of their anxiety, depression, and relationship satisfaction [23]. Integrating caregivers’ ongoing unmet needs such as those identified in our study into survivorship care can address their concerns and prevent these negative outcomes. Addressing the concerns and needs of caregivers as an aspect of survivorship care may reduce distress among patients and improve quality of life.

In spite of most patients and caregivers in our study being highly satisfied with information supplied by and resources identified by their HCPs, they still reported informational needs that remained unaddressed. Notably, some stakeholders wish to receive a written document including information about what to expect after treatment and extensive and up-to-date information on specific topics, which largely resembles a Survivorship Care Plan (SCP) as was proposed by the NAM since 2006 [11]. However, other patients and caregivers did not describe a clear need for additional resources or desire for more information. In this wide range of needs and preferences, a “one size fits all” approach may not be most effective nor efficient. This might explain why previous trials assessing the effectiveness of SCPs failed to identify benefits in unselected populations, including samples of American [13] and Dutch [16, 17] gynecological cancer patients. Though women in the latter trial only included endometrial and ovarian cancer, previous analyses suggest that patients’ benefit of SCPs is indeed heterogenous [24, 25]. Ideally, survivorship resources should be allocated to those with highest neccesity and be updated over time. This highlights a need for screening for informational and other needs as an important part of survivorship care, and a necessary step in the development of individualized SCPs. Future SCP effectiveness trials should focus on individualized SCPs, particularly when assessed in heterogenous patient populations such as in gynecological oncology.

As most patients and caregivers did not indicate a clear preference for either written or online SCPs, an online, patient-centered application including tailored information for those with specific needs could be a solution that fits the needs of all stakeholders. A written leaflet including more general information could complement the online tool, or even replace it for those with minimal information needs. Further, patients and caregivers who were interested in an SCP indicated that they would like to receive one during the first follow-up visit after the end of treatment, and prefer a conversation accompanied with it, as opposed to generation of a document alone. An important finding of this study is that patients and caregivers do not indicate a strong preference for the person leading this conversation. Conversely, HCPs in our study believe that patients prefer their treating oncologist to provide survivorship care planning. However, they also recognize that this may not be feasible in their practice due to increasing clinical burdens and lack of time. Previous studies also found that lack of time was cited as the greatest barrier to implementation of SCPs [26, 27]. While oncologists buy into the concepts of survivorship care planning, the suggestions from providers in our study offer potential ways to address systematic implemention including personalization of care plans to individual patients, inclusion of a dedicated support staff to facilitate discussions, and creation of a prompt list to initiate the discussion using careful but clear communication strategies. Our study supports that patients and caregivers may be amenable to receive SCPs by other members of the care team besides the oncologist, depending on the clinical practices’ logistics and feasibility.

This study includes a variety of gynecological cancer types and stages, caregiver types, and gynecologic oncology HCP. Even though our sample was reasonably heterogenous, we reached data saturation for all groups. Our qualitative data allowed for assessment of unique individual and heterogenous experiences of stakeholders. Our findings provide detailed in-depth descriptions of the various perspectives in this field and enrich the limited literature available. However, a limitation of this study includes the use of a single medical center to recruit participants, serving patients with a relatively high socio-economic status and few ethnic minorities. Further, only one patient and her caregiver were clearly dissatisfied with care at our center, which is not in coherence with literature showing much higher proportions of dissatisfaction with care [28, 29], resulting in potentially biased descriptions of concerns and preferences.

## Conclusion

In conclusion, patients and caregivers in this study endorsed the need for personalized, tailored survivorship care planning starting near the end of treatment. Patients with ovarian cancer reported qualitatively different experiences and desires as patient with non-ovarian gynecological cancers, indicating these groups may require distinct forms of care planning. HCPs require assistance in starting sensitive conversations at the end of treatment, but are open to providing individualized SCPs to their patients within the context of the entire team. These qualitative findings provide a description of the self-reported needs of multiple stakeholders, highlight barriers and opportunties to address survivorship needs within the gynecology oncology clinic, and can be used to support the development of patient-centered survivorship care planning interventions.

## Electronic supplementary material

ESM 1 (DOCX 44 kb)
