# Supplementary material for: Survivorship care planning in gynecologic oncology—perspectives from patients, caregivers, and health care providers
Source: J Cancer Surviv. 2018 Sep 12;12(6):762–74. doi: 10.1007/s11764-018-0713-9 (PMC6244937; doi:10.1007/s11764-018-0713-9)
Supplement: Supplementary file 1 — (DOCX 44 kb) [file 11764_2018_713_MOESM1_ESM.docx]

**Appendix I:** Themes and codes of the health care provider focus group

| **Challenges in post-treatment care** |
| --- |
| **Struggling to Provide Care at End of Treatment** |
| Struggling to find necessary resources for patients |
| Challenges of communication between nurse practitioners and doctors |
| Feeling alone without resources |
| Challenges in communicating about survivorship (tension between being honest and being cruel) |
| Approaching with cautious optimism and genuine emotion |
| Struggling with what to say |
| **Recurrence and Uncertainty** |
| Fear of recurrence |
| Uncertainty about future |
| Anxiety about stopping treatment |
| Following (being influenced by) patient’s lead |
| Patients needing you to be realistically optimistic |
| Moving forward in face of recurrence/ uncertainty |
| Reinforcing patients should live life in spite of fear and uncertainty |
| Living but dead |
| **Reviewing Patients’ Needs** |
| Providing surveillance data |
| Providing a review of symptoms specific to each patient |
| Provide alarms to patients who are cured |
| Patients researching online |
| **Enhancing Post Treatment Life** |
| Reinforcing patients should live life in spite of fear and uncertainty |
| Support quality of life |
| Not wanting to scare patients/ improve quality of life |
| Provide calm to patients who are hypervigilant |
| Wanting reassurance that others experience similar problems |
| Developing a strategy for moving forward |
| Not delaying life |
| Possibility to alleviate terror |
| Making patients feel heard |
| Discussing physical attractiveness |
| Demonstrating love for patients |
| **Need for survivorship care plan (SCP) and resources** |
| **Need for Planning and Resources** |
| Providers’ preferences for want to accomplish in a survivorship visit |
| Challenges in communicating about survivorship (tension between being honest and being cruel): |
| Valuing patient-provider relationship as SCP facilitator |
| Need for tailored SCPs |
| Survivorship planning related to type of cancer and SCP |
| Need for SCP (most common issues/ percentages) |
| Need for SCP |
| reassurance |
| Sexual health |
| Support groups |
| Need for rehab program |
| **Providing Care at End of Treatment** |
| Suggesting diet, exercise, and attitude for promoting health |
| Providing sexual health/intimacy counseling and treatment |
| Contacting others for referrals |
| Documenting care with follow-up plan |
| Discussing beauty and loss of hair |
| Sharing what to expect and normalizing |
| **Survivorship Care Plan (SCP)** |
| Not providing formal SCPs |
| Need for SCP |
| Follow-up plan |
| Written information |
| Supplement to a conversation |
| Creating a conversation starter |
| Referencing what to look out for and when to come in to be seen |
| Providing resources for how to get back to normal |
| Providing information on what to expect |
| Wanting a physical document |
| Providing health promotion information |
| Designing a reasonable plan to get buy-in/ follow-up plan |
| Care planning not disease specific |
| Survivorship planning related to type of cancer and SCP |
| **Barriers to providing survivorship care plans** |
| **Barriers to SCPs** |
| Challenges in communicating about survivorship (tension between being honest and being cruel) |
| Barrier of time |
| Not wanting to open up difficult needs |
| **Preferences for provider of SCP** |
| Nurse/ oncologist/ anybody with time |
| **Logistical concerns** |
| Standardized list of prompts / Screening tools |
| Logistics of providing care plan |
